# Supplementary material for: TMEM87a/Elkin1, a component of a novel mechanoelectrical transduction pathway, modulates melanoma adhesion and migration
Source: eLife. 2020 Apr 1;9:e53308. doi: 10.7554/eLife.53308 (PMC7173973; doi:10.7554/eLife.53308)
Supplement: Figure 1—source data 1. [file elife-53308-fig1-data1.docx]

| **Substrate** | **PDMS** | | **LM511** | |
| --- | --- | --- | --- | --- |
| **Cells** | **WM115** | **WM266-4** | **WM115** | **WM266-4** |
| **Latency** | 3.0 ± 0.6 ms  (n=18) | 1.7 ± 0.3 ms  (n = 42) | 2.0 ± 0.6 ms  (n = 26) | 2.1 ± 0.3 ms  (n = 62) |
| **Activation time constant** | 1.5 ± 0.4 ms | 0.7 ± 0.2 ms | 1.5 ± 0.8 ms | 0.7 ± 0.1 ms |
| **Inactivation time constant (inactivating currents)** | 9.6 ± 4.4 ms  (n = 12) | 30 ± 11 ms  (n = 30) | 8.9 ± 3.5 ms  (n = 19) | 13.7 ± 3.7 ms  (n = 53) |
| **Percentage non-inactivating** | 33%  6/18 | 36%  15/42 | 27%  7/26 | 15%  9/62 |

**Figure 1- source data: Physiological properties of currents recorded in melanoma cells**

WM155 and WM266-4 cells were cultured on pillar arrays that were either uncoated (PDMS) or coated with LM511. For each group the mechanical latency, activation time constant (calculated from a mono-exponential fit of current activation) and inactivation time constant (time constant calculated from a mono-exponential fit of the current inactivation, when relevant) are shown. Data are displayed as mean ± s.e.m. In addition, the percentage of currents that were non-inactivating is presented.
