# Supplementary material for: TMEM87a/Elkin1, a component of a novel mechanoelectrical transduction pathway, modulates melanoma adhesion and migration
Source: eLife. 2020 Apr 1;9:e53308. doi: 10.7554/eLife.53308 (PMC7173973; doi:10.7554/eLife.53308)
Supplement: Figure 3—source data 1. [file elife-53308-fig3-data1.docx]

| **HEK293-P1KO** | ***hs*Elkin1 iso1** | ***hs*Elkin1 iso3** |
| --- | --- | --- |
| **Latency** | 1.2 ± 0.7 ms  (n=25) | 1.9 ± 0.2 ms  (n = 39) |
| **Activation time constant** | 0.4 ± 0.09 ms | 0.9 ± 0.4 ms |
| **Inactivation time constant (inactivating currents)** | 37.8 ± 32 ms  (n = 20) | 33 ± 16 ms  (n = 30) |
| **Fraction non-inactivating** | 20%  (5/25) | 20%  (8/39) |

**Figure 3- source data: Physiological properties of currents recorded in HEK-293 P1KO cells**

HEK-293 P1KO cells expressing Elkin1 variants were cultured on pillar arrays. For each group the mechanical latency, activation time constant (calculated from a mono-exponential fit of current activation) and inactivation time constant (time constant calculated from a mono-exponential fit of the current inactivation, when relevant) are shown. Data are displayed as mean ± s.e.m. In addition, the percentage of currents that were non-inactivating is presented.
