# Supplementary material for: TMEM87a/Elkin1, a component of a novel mechanoelectrical transduction pathway, modulates melanoma adhesion and migration
Source: eLife. 2020 Apr 1;9:e53308. doi: 10.7554/eLife.53308 (PMC7173973; doi:10.7554/eLife.53308)
Supplement: Figure 4—source data 1. [file elife-53308-fig4-data1.docx]

| **HEK293-P1KO** | ***mm*Elkin1**  **iso1** | ***mm*Elkin1 iso1 F271L, N292G** |
| --- | --- | --- |
| **Latency** | 3.5 ± 1.3 ms  (n = 18) | 1.5 ± 0.3 ms  (n = 40) |
| **Activation time constant** | 1.4 ± 0.3 ms | 1.1 ± 0.3 ms |
| **Inactivation time constant (inactivating currents)** | 24 ± 13 ms  (n = 7) | 56 ± 27 ms  (n = 27) |
| **Fraction non-inactivating** | 61%  (11/18) | 32.5%  (13/40) |

**Figure 4- source data: Physiological properties of currents recorded in HEK-293 P1KO cells**

HEK-293 P1KO cells expressing Elkin1 variants were cultured on pillar arrays. For each group the mechanical latency, activation time constant (calculated from a mono-exponential fit of current activation) and inactivation time constant (time constant calculated from a mono-exponential fit of the current inactivation, when relevant) are shown. Data are displayed as mean ± s.e.m. In addition, the percentage of currents that were non-inactivating is presented.
