# Supplementary material for: TMEM87a/Elkin1, a component of a novel mechanoelectrical transduction pathway, modulates melanoma adhesion and migration
Source: eLife. 2020 Apr 1;9:e53308. doi: 10.7554/eLife.53308 (PMC7173973; doi:10.7554/eLife.53308)
Supplement: Figure 6—source data 1. [file elife-53308-fig6-data1.docx]

**Figure 6: Elkin1-KO cells exhibit decreased migration on 2D and *quasi*-1D substrates**

**Figure 6C,D**

| **2D LM511 Track mean speed (µm/min)** | | |
| --- | --- | --- |
|  | **WT** | **KO** |
| N (tracks)  Mean ± s.e.m.  Mean CI (95%)  Median  Quartiles  Median CI (95%) | 246  0.383 ± 0.011  0.361 – 0.404  0.356  0.256 – 0.467  0.319 – 0.392 | 340  0.275 ± 0.009  0.257 – 0.294  0.240  0.173 – 0.330  0.211 – 0.240 |
| **2D PLL Track mean speed (µm/min)** | | |
|  | **WT** | **KO** |
| N (tracks)  Mean ± s.e.m.  Mean CI (95%)  Median  Quartiles  Median CI (95%) | 240  0.270 ± 0.007  0.256 – 0.283  0.263  0.180 – 0.347  0.240 – 0.279 | 241  0.189 ± 0.005  0.180 – 0.199  0.180  0.120 – 0.240  0.170 – 0.180 |
| **2D LM511 Euclidean distance (µm)** | | |
|  | **WT** | **KO** |
| N (tracks)  Mean ± s.e.m.  Mean CI (95%)  Median  Quartiles  Median CI (95%) | 246  0.357 ± 0.016  0.326 – 0.388  0.298  0.182 – 0.486  0.266 – 0.337 | 340  0.267 ± 0.014  0.238 – 0.295  0.185  0.097 – 0.320  0.169 – 0.200 |
| **2D PLL Euclidean distance (µm)** | | |
|  | **WT** | **KO** |
| N (tracks)  Mean ± s.e.m.  Mean CI (95%)  Median  Quartiles  Median CI (95%) | 240  0.222 ± 0.012  0.200 – 0.245  0.182  0.095 – 0.292  0.165 – 0.206 | 241  0.169 ± 0.007  0.154 – 0.183  0.136  0.090 – 0.228  0.127 – 0.156 |

**Figure 6F**

| ***quasi*-1D LM511 Track mean speed (µm/min)** | | |
| --- | --- | --- |
|  | **WT** | **KO** |
| N (tracks)  Mean ± s.e.m.  Mean CI (95%)  Median  Quartiles  Median CI (95%) | 260  1.173 ± 0.027  1.120 – 1.227  1.140  0.84 – 1.44  1.08 – 1.26 | 275  0.938 ± 0.026  0.886 – 0.989  0.960  0.60 – 1.26  0.84 – 1.02 |
