# Supplementary material for: TMEM87a/Elkin1, a component of a novel mechanoelectrical transduction pathway, modulates melanoma adhesion and migration
Source: eLife. 2020 Apr 1;9:e53308. doi: 10.7554/eLife.53308 (PMC7173973; doi:10.7554/eLife.53308)
Supplement: Figure 7—source data 1. [file elife-53308-fig7-data1.docx]

**Source data, Figure 7 and figure 7 supplements**

**Figure 7 and figure 7- figure supplement 1: Elkin1-KO increases cell dissociation from organotypic spheroids**

Note: these values are taken from the same data sets. Where n = number of spheroids the data have been averaged for each spheroid (Figure 7), data are also presented for n = cells, where values have been calculated for all of the individual cells (not averaged for each spheroid)(Figure 7- figure supplement 1).

| **Spheroids: Number of dissociated cells** | | | |
| --- | --- | --- | --- |
| WT (averaged/ spheroid) | | | |
| Time (h) | 24 | 48 | 72 |
| N  Mean ± s.e.m.  Mean CI (95%)  Median  Quartiles  Median CI (95%) | 9 spheroids  75 ± 17  34 – 115  61  47 – 92  29 - 182 | 9 spheroids  177 ± 30  105 – 249  153  124 – 230  69 - 348 | 9 spheroids  324 ± 33  246 – 402  301  261 – 402  211 - 492 |
| KO (averaged/ spheroid) | | | |
| Time (h) | 24 | 48 | 72 |
| N  Mean ± s.e.m.  Mean CI (95%)  Median  Quartiles  Median CI (95%) | 9 spheroids  640 ± 89  436 – 845  654  405 – 845  392 - 852 | 9 spheroids  1239 ± 101  1006 – 1472  1409  974 – 1511  960 - 1549 | 9 spheroids  1752 ± 71  1589 – 1916  1820  1584 – 1913  1478 – 1913 |
| **Spheroids: Distance from spheroid (µm)** | | | |
| WT (averaged/ spheroid) | | | |
| Time (h) | 24 | 48 | 72 |
| N (spheroids)  Mean ± s.e.m.  Mean CI (95%)  Median  Quartiles  Median CI (95%) | 9  226.6 ± 22.48  173.5 - 279.8  216.6  197.9 - 249.3  130 - 356 | 9  216.0 ± 10.75  191.2 - 240.8  223.1  191.2 - 239.5  189 - 241 | 9  246.2 ± 9.345  224.7 - 267.8  253.8  218.2 - 272.2 |
| KO (averaged/ spheroid) | | | |
| Time (h) | 24 | 48 | 72 |
| N (spheroids)  Mean ± s.e.m.  Mean CI (95%)  Median  Quartiles  Median CI (95%) | 9 spheroids  255.9 ± 10.6  231.5 – 280.2  254.2  227.8 – 285.1  227.2 – 286.5 | 9 spheroids  336.6 ± 17.2  297 – 376.2  366.2  285.7 – 378.9  278.4 – 385.6 | 9 spheroids  418.9 ± 20.2  372.5 – 465.4  444.7  358.1 – 472.1  350 – 480.4 |
| WT (individual cells) | | | |
| N (cells)  Mean ± s.e.m.  Mean CI (95%)  Median  Quartiles  Median CI (95%) | 156  222 ± 7.5  207 – 237  205  174 – 242  194 - 216 | 394  228 ± 3.3  222 – 235  219  182 – 263  212 - 226 | 546  261 ± 3.2  255 – 267  252  207 – 302  240 - 262 |
| KO (individual cells) | | | |
| N (cells)  Mean ± s.e.m.  Mean CI (95%)  Median  Quartiles  Median CI (95%) | 3659  269 ± 1.7  266 – 273  257  197 – 326  253 - 261 | 9115  352 ± 1.3  350 – 355  346  252 – 444  343 - 349 | 12690  430 ± 1.5  427 – 433  417  296 – 550  413 – 421 |
| **Spheroids: Sphericity** | | | |
| WT (averaged/ spheroid) | | | |
| Time (h) | 24 | 48 | 72 |
| N (spheroids)  Mean ± s.e.m.  Mean CI (95%)  Median  Quartiles  Median CI (95%) | 9 spheroids  0.88 ± 0.02  0.84 – 0.92  0.89  0.86 – 0.92  0.79 – 0.94 | 9 spheroids  0.91 ± 0.004  0.90 – 0.92  0.90  0.90 – 0.92  0.90 – 0.92 | 9 spheroids  0.92 ± 0.005  0.90 – 0.93  0.91  0.91 – 0.93  0.90 – 0.94 |
| KO (averaged/ spheroid) | | | |
| Time (h) | 24 | 48 | 72 |
| N (spheroids)  Mean ± s.e.m.  Mean CI (95%)  Median  Quartiles  Median CI (95%) | 9 spheroids  0.83 ± 0.006  0.81 – 0.84  0.83  0.80 – 0.84  0.80 – 0.85 | 9 spheroids  0.87 ± 0.007  0.86 – 0.89  0.86  0.86 – 0.89  0.86 – 0.90 | 9 spheroids  0.90 ± 0.003  0.89 – 0.90  0.90  0.89 – 0.90  0.88 – 0.90 |
| WT (individual cells) | | | |
| N (cells)  Mean ± s.e.m.  Mean CI (95%)  Median  Quartiles  Median CI (95%) | 156  0.86 ± 0.006  0.85 – 0.87  0.88  0.80 – 0.91  0.85 – 0.89 | 394  0.90 ± 0.003  0.90 – 0.91  0.92  0.88 – 0.95  0.92 – 0.93 | 546  0.91 ± 0.002  0.90 – 0.91  0.92  0.89 – 0.94  0.92 – 0.93 |
| KO (individual cells) | | | |
| N (cells)  Mean ± s.e.m.  Mean CI (95%)  Median  Quartiles  Median CI (95%) | 3519  0.83 ± 0.001  0.82 – 0.83  0.84  0.78 – 0.90  0.84 – 0.84 | 8651  0.87 ± 0.001  0.87 – 0.88  0.89  0.83 – 0.93  0.89 – 0.89 | 11756  0.90 ± 0.001  0.90 - 0.90  0.91  0.87 – 0.94  0.91 – 0.92 |

**Figure 7- figure supplement 2: The effect of Elkin1 deletion on migration in 3D collagen gels**

| **Isolated cells: Mean track speed (µm/min)** | | |
| --- | --- | --- |
|  | **WT** | **KO** |
| N (tracks)  Mean ± s.e.m.  Mean CI (95%)  Median  Quartiles  Median CI (95%) | 3135  0.41 ± 0.003  0.40 – 0.42  0.39  0.29 – 0.51  0.39 – 0.40 | 1599  0.41 ± 0.005  0.40 – 0.42  0.39  0.28 – 0.54  0.38 – 0.40 |
| **Isolated cells: cell sphericity (averaged over whole track)** | | |
|  | **WT** | **KO** |
| N (tracks)  Mean ± s.e.m.  Mean CI (95%)  Median  Quartiles  Median CI (95%) | 3135  0.873 ± 0.001  0.870 – 0.876  0.898  0.843 - 0.924  0.896 - 0.900 | 1599  0.871 ± 0.002  0.868 - 0.874  0.888  0.840 – 0.910  0.885 – 0.891 |
| **Isolated cells: track straightness** | | |
|  | **WT** | **KO** |
| N (tracks)  Mean ± s.e.m.  Mean CI (95%)  Median  Quartiles  Median CI (95%) | 3135  0.39 ± 0.004  0.38 - 0.40  0.35  0.20 - 0.54  0.34 - 0.36 | 1599  0.35 ± 0.006  0.33 - 0.36  0.27  0.13 - 0.53  0.25 – 0.29 |
