# Supplementary material for: TMEM87a/Elkin1, a component of a novel mechanoelectrical transduction pathway, modulates melanoma adhesion and migration
Source: eLife. 2020 Apr 1;9:e53308. doi: 10.7554/eLife.53308 (PMC7173973; doi:10.7554/eLife.53308)
Supplement: Figure 8—source data 1. [file elife-53308-fig8-data1.docx]

**Figure 8- source data, unbinding forces**

| **Maximum unbinding force: Cell-substrate interactions (nN)** | | |
| --- | --- | --- |
|  | **WT** | **KO** |
| N (curves-technical)  Mean ± s.e.m.  Mean CI (95%)  Median  Quartiles  Median CI (95%) | 50  0.25 ± 0.01  0.22 – 0.27  0.24  0.17 – 0.29  0.20 – 0.27 | 52  0.40 ± 0.03  0.33 – 0.47  0.38  0.23 – 0.49  0.29 – 0.45 |
| **Maximum unbinding force: Cell-cell interactions (pN)** | | |
|  | **WT** | **KO** |
| N (curves)  Mean ± s.e.m.  Mean CI (95%)  Median  Quartiles  Median CI (95%) | 83  307 ± 13  282 – 333  291  219 – 386  252 - 326 | 70  229 ± 14  200 – 258  185  149 – 290  168 - 229 |
