# Supplementary material for: TMEM87a/Elkin1, a component of a novel mechanoelectrical transduction pathway, modulates melanoma adhesion and migration
Source: eLife. 2020 Apr 1;9:e53308. doi: 10.7554/eLife.53308 (PMC7173973; doi:10.7554/eLife.53308)
Supplement: Supplementary file 2. [file elife-53308-supp2.docx]

| **Key Resources Table** | | | | |
| --- | --- | --- | --- | --- |
| **Reagent type (species) or resource** | **Designation** | **Source or reference** | **Identifiers** | **Additional information** |
| cell line (*Homo-sapiens*) | WM266-4 | ATCC | RRID:CVCL_2765 |  |
| cell line (*Homo-sapiens*) | WM266-4 3B6 | This paper | Clonal isolate post CRISPR/Cas9 treatment | Elkin1-WT clone |
| cell line (*Homo-sapiens*) | WM266-4 3E9 | This paper | Clonal isolate post CRISPR/Cas9 treatment | Elkin1-WT clone |
| cell line (*Homo-sapiens*) | WM266-4 3C6 | This paper | Clonal isolate post CRISPR/Cas9 treatment | Elkin1-KO clone |
| cell line (*Homo-sapiens*) | WM266-4 3C6-Elkin1-iso3-GFP | This paper | 3C6 cells stably expressing Elkin1-iso3 and GFP  (linearised plasmid 52) |  |
| cell line (*Homo-sapiens*) | WM266-4 3C6-Elkin1-iso3-L210F-GFP | This paper | 3C6 cells stably expressing Elkin1-iso3-L210F and GFP  (linearised plasmid 64) |  |
| cell line (*Homo-sapiens*) | WM266-4 3D6 | This paper | Clonal isolate post CRISPR/Cas9 treatment | Elkin1-KO clone |
| cell line (*Homo-sapiens*) | WM115 | ATCC | RRID:CVCL_0040 |  |
| cell line (*Homo-sapiens*) | A375 | ATCC | RRID:CVCL_0132 |  |
| cell line (*Homo-sapiens*) | A375- 1B6 | This paper | Clonal isolate post CRISPR/Cas9 treatment | Elkin1-WT clone |
| cell line (*Homo-sapiens*) | A375- 1B12 | This paper | Clonal isolate post CRISPR/Cas9 treatment | Elkin1-KO clone |
| cell line (*Homo-sapiens*) | HEK293T P1KO | Lukacs et al., 2015 | HEK293T cells with frameshift mutation in *PIEZO1* | Gift from A. Patapoutian |
| Cell line  (*Mus musculus*) | N2a *^Piezo1-/-^* | Moroni et al., 2018 | Clonal isolate, Neuro2A edited to delete *PIEZO1* |  |
| Transduced construct (*Homo-sapiens)* | pRRLSIN.cPPT.PGK-GFP.WPRE | Didier Trono/Inder Verma, | RRID:Addgene_12252 | Gift from I. Alexander |
| Transfected construct (*Homo-sapiens*) | psPAX2 | Didier Trono | RRID:Addgene_12260 | Gift from I. Alexander |
| Transfected construct (*Homo-sapiens*) | pMD2.G | Didier Trono | RRID:Addgene_12259 | Gift from I. Alexander |
| Commercial assay or kit | Pierce Mass Spec Sample Prep Kit for Cultured Cells | ThermoFisher | 84840 |  |
| Commercial assay or kit | RNEasy kit | Qiagen | 74104 |  |
| Chemical compound | EZ-link Sulfo-NHS-LC-LC-biotin | ThermoFisher | 21338 | 2.5 mg/ml |
| Chemical compound | NeutrAvidin Ultralink Resin | ThermoFisher | 53150 |  |
| Antibody | Anti-GFP (rabbit polyclonal) | Sigma Aldrich | SAB4301138 | 1:1000 |
| Antibody | HRP-linked anti-rabbit IgG (goat polyclonal) | Cell Signalling technologies | 7074 | 1:1000 |
| Commercial assay or kit | BLOCK-iT™ Pol II miR RNAi Expression Vector | Invitrogen | K493600 |  |
| Sequence-based reagent | Elkin1-iso1 forward | This paper | PCR primer | 5’ ATGGCGGCGGCTGCGTGGC |
| Sequence-based reagent | Elkin1-iso3 forward | This paper | PCR primer | 5’ ATGGCACATTCCGATACCGT |
| Sequence-based reagent | Elkin1-1/3 reverse | This paper | PCR primer | 5’ TTACTCCATTTTGGACCTTTC |
| Sequence-based reagent | Elkin1 – siRNA1 | This paper | DNA sequence for creating miR RNAi construct | 5’ ACCTTGTGACCTGTCTTTGAA |
| Sequence-based reagent | Elkin1 – siRNA2 | This paper | DNA sequence for creating miR RNAi construct | 5’ CTTCCTGGGAATGCTTGAGAA |
| Sequence-based reagent | Elkin1 – siRNA3 | This paper | DNA sequence for creating miR RNAi construct | 5’ TGGCAGCATCCATTGTGTTTA |
| Sequence-based reagent | Piezo1 – siRNA1 | This paper | DNA sequence for creating miR RNAi construct | 5’ ACTCATCAAGTGGCTGTACCT |
| Sequence-based reagent | Piezo1 – siRNA2 | This paper | DNA sequence for creating miR RNAi construct | 5’ CTGCTTCTACCTGCTGCTCTT |
| Sequence-based reagent | Piezo1 – siRNA3 | This paper | DNA sequence for creating miR RNAi construct | 5’ GCGTCTTCCTTAGCCATTACT |
| Transfected construct (*Homo-sapiens*) | pSpCas9n(BB)-2A-GFP plasmid | Ran et al., 2013 | RRID:Addgene_48140 | Gift from Feng Zhang |
| Commercial assay or kit | Illustra Genomic Prep Mini Spin kit | GE Life Sciences | 28904275 |  |
| recombinant DNA reagent | Plasmid 46 | This paper |  | Plasmid encoding hsElkin1-iso1 with IRES GFP |
| recombinant DNA reagent | Plasmid 47 | This paper |  | Plasmid encoding hsElkin1-iso3 with IRES GFP |
| recombinant DNA reagent | Plasmid 51 | This paper |  | Plasmid encoding hsElkin1-iso1-GFP fusion construct |
| recombinant DNA reagent | Plasmid 52 | This paper |  | Plasmid encoding hsElkin1-iso3-GFP fusion construct |
| recombinant DNA reagent | Plasmid 12 | This paper |  | Plasmid encoding mmElkin1 with IRES GFP |
| recombinant DNA reagent | Plasmid 64 | This paper |  | Plasmid encoding hsElkin1-iso3-L210F with IRES GFP |
| recombinant DNA reagent | Plasmid 65 | This paper |  | Plasmid encoding hsElkin1-iso3-G230N with IRES GFP |
| recombinant DNA reagent | Plasmid 7879 | This paper |  | Plasmid encoding mmElkin1-F271L/N292G with IRES GFP |
| recombinant DNA reagent | Plasmid 122 | This paper |  | Plasmid encoding mmElkin1- GFP fusion |
| recombinant DNA reagent | Plasmid 123 | This paper |  | Plasmid encoding mmElkin1- F271L/N292G-GFP fusion |
| recombinant DNA reagent | Plasmid 75 | This paper |  | Plasmid encoding hsElkin1-iso3-delta 0-108 GFP |
| recombinant DNA reagent | Plasmid 76 | This paper |  | Plasmid encoding hsElkin1-iso3-delta 0-146 GFP |
| recombinant DNA reagent | Plasmid 77 | This paper |  | Plasmid encoding hsElkin1-iso3-delta 0-208 GFP |
| Sequence-based reagent | CRISPR-CAS9 gene editing  Intron7-1 | This paper | DNA sequence to encode gRNA for CRISPR/Cas9 editing | 5’ CACCGAGGACACAGCATGAACTATG |
| Sequence-based reagent | CRISPR-CAS9 gene editing  Intron7-1 complement | This paper | DNA sequence to encode gRNA for CRISPR/Cas9 editing | 5’ AAACCATAGTTCATGCTGTGTCCTC |
| Sequence-based reagent | CRISPR-CAS9 gene editing  Intron7-2 | This paper | DNA sequence to encode gRNA for CRISPR/Cas9 editing | 5’ CACCGCCTCCACCATAGTCTGAAG |
| Sequence-based reagent | CRISPR-CAS9 gene editing  Intron7-2 complement | This paper | DNA sequence to encode gRNA for CRISPR/Cas9 editing | 5’ AAACCTTCAGACTATGGTGGAGGC |
| Sequence-based reagent | CRISPR-CAS9 gene editing  Exon9-1 | This paper | DNA sequence to encode gRNA for CRISPR/Cas9 editing | 5’ CACCGCAGCTTTCTCAAGCATTCCC |
| Sequence-based reagent | CRISPR-CAS9 gene editing  Exon9-1 complement | This paper | DNA sequence to encode gRNA for CRISPR/Cas9 editing | 5’ AAACGGGAATGCTTGAGAAAGCTGC |
| Sequence-based reagent | CRISPR-CAS9 gene editing  Exon9-2 | This paper | DNA sequence to encode gRNA for CRISPR/Cas9 editing | 5’ CACCGTTTCAGAATATCCGATACAA |
| Sequence-based reagent | CRISPR-CAS9 gene editing  Exon9-2 complement | This paper | DNA sequence to encode gRNA for CRISPR/Cas9 editing | 5’ AAACTTGTATCGGATATTCTGAAAC |
| Peptide, recombinant protein | LM511 | BioLamina | LN511 | Purified recombinant laminin |
| Peptide, recombinant protein | LM411 | BioLamina | LN411 | Purified recombinant laminin |
| Peptide, recombinant protein | LM211 | BioLamina | LN211 | Purified recombinant laminin |
| Peptide, recombinant protein | LM111 | BioLamina | LN111 | Purified recombinant laminin |
